# Methyl Chavicol and Its Synthetic Analogue as Possible Antioxidant and Antilipase Agents Based on the *In Vitro* and In Silico Assays

**DOI:** 10.1155/2018/2189348

**Published:** 2018-04-11

**Authors:** Bruna Celeida Silva Santos, Andressa Soares Pires, Célia Hitomi Yamamoto, Mara Rubia Costa Couri, Alex Gutterres Taranto, Maria Silvana Alves, Ana Lúcia dos Santos de Matos Araújo, Orlando Vieira de Sousa

**Affiliations:** ^1^Graduate Program in Pharmaceutical Sciences, Faculty of Pharmacy, Federal University of Juiz de Fora, Campus Universitário, São Pedro, 36036-900 Juiz de Fora, MG, Brazil; ^2^Department of Pharmaceutical Sciences, Faculty of Pharmacy, Federal University of Juiz de Fora, Campus Universitário, São Pedro, 36036-900 Juiz de Fora, MG, Brazil; ^3^Department of Chemistry, Institute of Exact Sciences, Federal University of Juiz de Fora, Campus Universitário, São Pedro, 36036-900 Juiz de Fora, MG, Brazil; ^4^Laboratory of Medicinal Pharmaceutical Chemistry, Federal University of São João Del-Rei, Campus Centro Oeste, Rua Sebastião Gonçalves Coelho, 400, Chanadour, 35501-296 Divinópolis, MG, Brazil

## Abstract

This study investigated the *in vitro* and in silico biological properties of the methyl chavicol (MC) and its analogue 2-[(4-methoxyphenyl)methyl]oxirane (MPMO), emphasizing the antioxidant and antilipase effects. MPMO was synthesized from MC that reacted with *meta*-chloroperbenzoic acid and, after separation and purification, was identified by ^1^H and ^13^C NMR and GC-MS. The antioxidant activity was investigated by DPPH, cooxidation *β*-carotene/linoleic acid, and thiobarbituric acid assays. With the use of colorimetric determination, the antilipase effect on the pancreatic lipase was tested, while the molecular interaction profiles were evaluated by docking molecular study. MC (IC_50_ = 312.50 ± 2.28 *μ*g/mL) and MPMO (IC_50_ = 8.29 ± 0.80 *μ*g/mL) inhibited the DPPH free radical. The inhibition of lipid peroxidation (%) was 73.08 ± 4.79 and 36.16 ± 4.11 to MC and MPMO, respectively. The malonaldehyde content was significantly reduced in the presence of MC and MPMO. MC and MPMO inhibited the pancreatic lipase in 58.12 and 26.93%, respectively. MC and MPMO (−6.1 kcal·mol^−1^) produced a binding affinity value lower than did diundecylphosphatidylcholine (−5.6 kcal·mol^−1^). These findings show that MC and MPMO present antioxidant and antilipase activities, which may be promising molecular targets for the treatment of diseases associated with oxidative damage and lipid metabolism.

## 1. Introduction

The imbalance between “prooxidant” and “antioxidant” chemical species produces oxidative stress, which causes lipidic peroxidation, aggression to proteins, and damage to DNA and RNA and triggers mechanisms associated with inflammatory, cardiovascular, and neuro-degenerative diseases; allergies; accelerated aging; hemorrhage; cataracts; immunological dysfunctions; and cancer [1, 2]. Among these disorders, the metabolic syndrome includes risk factors such as visceral obesity, endothelial dysfunction, dyslipidemia, and hypertension and is related to the development of type 2 diabetes mellitus with a high cardiovascular risk and mortality [3]. In addition, obesity and type 2 diabetes mellitus promote the increase of disease due to oxidative damage to proteins, lipids, DNA, and inflammatory process with generation of free radicals (FR) and deficiency in cell detoxification and repairs of damaged molecules [4]. In these pathways of metabolism, lipid peroxidation (LPO) is one of the triggered processes, since it forms lipid hydroperoxide by the incorporation of molecular oxygen to one of the polyunsaturated fatty acids. However, antioxidants can prevent the formation of FR or inhibit auto-oxidation, while the antilipase agents, such as orlistat, used in the treatment of obesity, are able to inhibit digestive lipases and reduce the absorption of fats from food, reducing cardiovascular risks [2, 5, 6]. These aspects interact with each other, and the search for new drugs that are capable of inhibiting oxidative and disease-associated mechanisms may be a great strategy for the treatment of different pathologies.

Methyl chavicol (MC), chemically known as 1-methoxy-4-prop-2-enylbenzene, estragole, or *p*-allylanisole, is a special metabolite belonging to the class of phenylpropanoids found in essential oils of medicinal and food plants [7]. The chemical structure consists of a benzene ring in the presence of a methoxy group (–OCH_3_) and a propenyl (–CH_3_CHCH_2_) at the 1 and 4 positions, respectively. The insecticidal activity of *Ocimum* spp. essential oils was attributed to this constituent against *Anopheles braziliensis*, a transmitter of malaria, dengue, and yellow fever [8]. This compound blocks voltage-activated sodium channels [9], and the anti-inflammatory activity is due to inhibition of leukocyte migration and stimulation of macrophages phagocytosis [10]. Pattnaik et al. [11] also revealed that MC showed a weak antimicrobial activity and the essential oil of *Ocimum basilicum* was cytotoxic against tumor cell lines such as Caco2 (colon cancer), HepG2 (hepatocellular cancer), and MCF-7 (breast adenocarcinoma).

Elevated doses of MC have hepatocarcinogenic potential, and the carcinogenicity is linked to 1′-hydroxy-methyl chavicol, a genotoxic metabolite catalyzed by cytochromes P4501A2 and P4502A6 [12–15]. In addition, the phase I metabolism includes O-demethylation, epoxidation, and 3′-hydroxylation reactions with formation of 4-allylphenol, methyl chavicol-2′, 3′-oxide, and 3′-hydroxyanethole, in this order [16–21]. The sulfonation reaction of 1′-hydroxy-methyl chavicol generates a carcinogenic metabolite, which is capable of reacting with DNA [14, 19, 22, 23].

As described above, biological properties of essential oils of medicinal and food plants have been attributed to MC, and the oxidative processes involve different mechanisms and pathological responses. In this sense, the understanding of antioxidant actions of promising compounds has been a strategy for the development of new therapeutic options for the treatment of metabolic disorders. Based on this principle, the present study aimed to synthesize an analogue from MC and evaluate the antioxidant activity and the inhibitory capacity on the pancreatic lipase using *in vitro* and in silico methods.

## 2. Materials and Methods

### 2.1. Chemicals and Reagents

The analytical products used for the development of this study were as follows: methyl chavicol (≥93.63%), *meta*-chloroperbenzoic acid (≥77%), and deuterated chloroform (CDCl_3_ 99.8%) (Sigma-Aldrich®); hexane (≥98%), ethyl ether (≥98%), chloroform (≥99%), hydrochloric acid (≥37%), methanol (≥98%), dichloromethane (≥98%), sulfuric acid (≥95%), sodium chloride (≥99.5%), sodium hydroxide (≥99%), pyridine (≥98%), acetic anhydride (≥95%), and potassium permanganate (≥98%) (Merck®); sodium bicarbonate (≥95%) and ethanol (≥99%) (BIOTEC®); iodine (≥95%) (Synth®); sodium sulfite (≥98%) and sodium thiosulfate (≥98%) (Reagen®); and anhydrous sodium sulfate (≥99%) (Quimex®).

### 2.2. Synthesis and Characterization of 2-[(4-Methoxyphenyl)methyl]oxirane (MPMO)

**Scheme 1:**
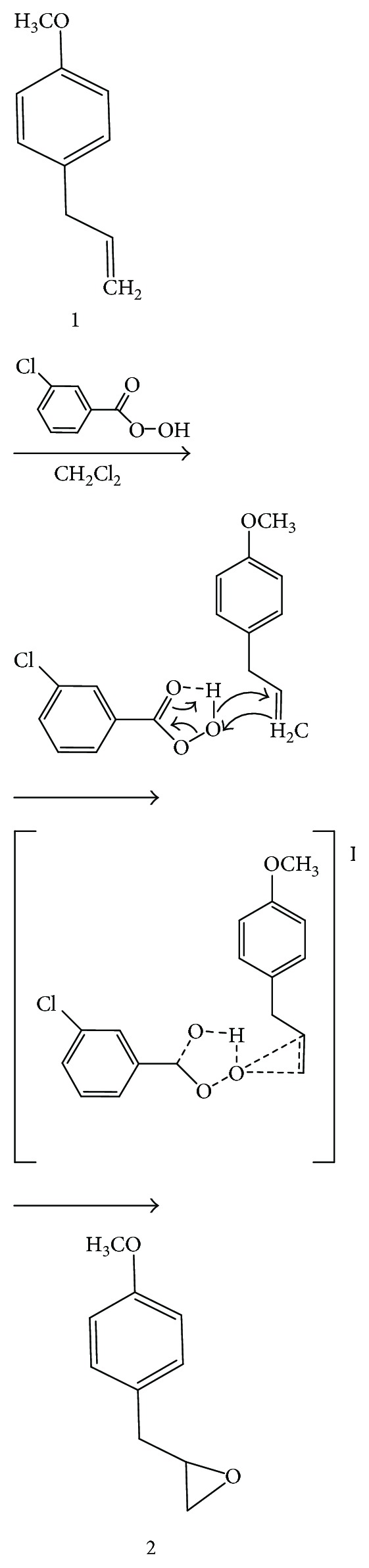
Oxidation reaction of methyl chavicol to obtain 2-[(4-methoxyphenyl)methyl]oxirane.

Methyl chavicol (**1**) (0.674 mmol/mL in dichloromethane) was reacted with *meta*-chloroperbenzoic acid (MCPBA) (0.35 mmol/mL in dichloromethane) for 40 minutes at temperature of 0°C (ice bath) and maintained at room temperature for 24 hours. After this time, 60 mL of 10% sodium sulfite (in water) was added to the reaction with stirring for one hour to separate the aqueous and organic phases. The aqueous phase was treated with dichloromethane, and, at the end of the separation, the organic phase was combined and washed with 5% sodium bicarbonate, saturated with sodium chloride solution, and subjected to anhydrous sodium sulfate to remove water residues. The solvent was evaporated, and the product was purified using silica gel chromatography column (70–230 mesh ASTM; Sigma-Aldrich) eluted in hexane/ethyl acetate (8 : 2). The yield of this reaction was 75% (Scheme 1).

^1^H and ^13^C nuclear magnetic resonance spectra of MC (Supplementary 1 and 2) and MPMO (Supplementary 4 and 5) were obtained at 500 MHz and 75 MHz, respectively, on a Bruker Avance DRX/500 spectrometer. As an internal reference, tetramethylsilane (TMS) or the residual hydrogen of the deuterated solvent was used. The chemical shift values (*δ*) were reported in parts per million (ppm) and the coupling constants (*J*) in hertz (Hz). The peak areas were acquired by electronic integration and their multiplicities described as follows: s = singlet; D = doublet; T = triplet; Tdd = triplet of double doublet; Dd = double doublet; Ddd = double doublet; and M = multiplet. MC spectral data were reported in Supplementary 1, 2, and 3 as described by the manufacturer.

MC and MPMO were analyzed by gas chromatography coupled to mass spectroscopy (GC-MS). The substances were diluted in 1% ethyl acetate (v/v), and 1.0 *μ*L was injected with flow division (1 : 20) into a gas chromatograph, model Shimadzu® GCMS-QP2010 Plus, capillary column type Rtx-5 (5% phenyl, 95% dimethylpolysiloxane). Helium was used as entrainment gas with a flow rate of 1.0 mL/min. The temperature was programmed from 60 to 240°C at a heating rate of 8°C/min. The mass detector was operated in the electron ionization mode (70 eV). The percentage composition of the synthesized products was obtained by normalization and integration of the peak areas.

For the chromatography column, silica gel 60G 0.063–0.200 mm (70–230 mesh ASTM, Sigma-Aldrich) was used, while for thin-layer chromatography (TLC), precoated alumina plates F_254_ (Sigma-Aldrich) and solvent systems containing hexane/ethyl acetate (9 : 1, 8 : 2, 7 : 3, 6 : 4, and 1 : 1) were used. Ultraviolet (UV) lamp at 254 nm and iodine vapors were used as developers.

### 2.3. DPPH Radical Sequestration Method

The antioxidant activity was determined by the 2,2-diphenyl-1-picryl-hydrazyl (DPPH) method as described by Mensor et al. [24]. From the stock solutions (750 mg/mL) of MC and MPMO and 3,5-di-*tert*-butyl-4-hydroxy toluene (BHT, 1 mg/mL) in ethanol PA, dilutions were prepared to obtain different concentrations. 2.5 mL was transferred, in triplicate, to test tubes, followed by addition of 1 mL of DPPH solution (0.03 mM). The antioxidant capacity was determined by reaction kinetics in the categories: rapid (reaction time < 30 minutes), medium (reaction time > 30 and <60 minutes), and slow (reaction time > 60 minutes) kinetics. The absorbances were plotted between zero time and 210 minutes (*t*_0_, *t*_15_, *t*_30_, *t*_45_, *t*_60_, *t*_75_, *t*_90_, *t*_120_, *t*_150_, *t*_180_, and *t*_210_) in an interval of 15 minutes [25]. After this time, the ability of the samples to reduce DDPH to 2,2-diphenyl-1-picryl hydrazine was observed by spectrophotometry (Shimadzu, UV-1800®) at 518 nm [24]. The blank (samples and BHT) consisted of 2.5 mL of solutions and 1.0 mL of ethanol. The negative control was composed of 2.5 mL of ethanol and 1.0 mL of DPPH solution, whose auto-zero was only performed with ethanol. From the absorbances (Abs), the percentage of antioxidant activity (% AA) was determined using the following equation:

(1) $$\begin{array}{c} \%AA=100-\frac{{Abs}_{sample}–{Abs}_{sample\;blank}}{{Abs}_{control}–{Abs}_{control\;blank}}\times 100. \end{array}$$

After linear regression analysis by least-squares method, the half maximal inhibitory concentration (IC_50_) was determined.

### 2.4. Cooxidation of the *β*-Carotene/Linoleic Acid Method

The antioxidant activity of MC and MPMO was determined by the cooxidation *β*-carotene/linoleic acid method described by Koleva et al. [26]. One milliliter of *β*-carotene (0.2 mg/mL in chloroform), 25 *μ*L of linoleic acid, and 200 mg of Tween 40 were placed into a rotavaporation flask. After that, the solvent was removed, and 50 mL of distilled water was slowly added, under constant stirring with bubbling oxygenation, to form an emulsion. In a microplate, 30 *μ*L of the samples and BHT (positive control) at 25 *μ*g/mL were added, in triplicate, followed by 250 *μ*L of the emulsion. The negative control was composed of 30 *μ*L of ethanol and 250 *μ*L of the emulsion. The blank was composed of 280 *μ*L of ethanol. The assay consisted of microplate readings between zero and 105 minutes (*t*_0_, *t*_15_, *t*_30_, *t*_45_, *t*_60_, *t*_75_, *t*_90_, and *t*_105_) at 15-minute intervals after incubation in an oven at 50°C. Absorbances were measured on a microplate reader (ThermoPlate®, TP-Reader) at 492 nm. The graph of the decay absorbances (Abs) as a function of time was elaborated, and the percentage of inhibition of the lipid peroxidation (% *I*) was determined from the following equation:

(2) $$\begin{array}{c} \%I=100-\frac{{Abs}_{control}-{Abs}_{sample}}{{Abs}_{control}}\times 100, \end{array}$$

where Abs_control_ = Abs_*t*_0__ − Abs_*t*_105__ and Abs_sample_ = Abs_*t*_0__ − Abs_*t*_105__ · Abs_control_: negative control and Abs_sample_: MC, MPMO, and BHT.

### 2.5. Thiobarbituric Acid Method

The lipid peroxidation method using thiobarbituric acid as described by Zeb and Ullah [27], with modifications, was applied to determine the antioxidant activity. This test consists in the analysis of the malonaldehyde and derivative substances from lipid peroxidation through the detection of the chromogenic complex [28]. Homogenates were prepared with 25 g of low-fat ground beef, 17 mL of distilled water, and 7.5, 15, and 30 mg of the samples in 200 *μ*L of methanol. The homogenates were heated until the meat was cooked. After this procedure, distilled water was added to complete 100 mL, and the homogenate was mixed, transferred to amber vials, and stored under refrigeration. In triplicate, the test was performed with 500 mg of each homogenate, 50 *μ*L of 4% BHT in ethanol, 2.5 mL of 1% phosphoric acid, and 1.25 mL of 1% thiobarbituric acid in 0.05 M sodium hydroxide. The tubes were boiled during 15 minutes, followed by cooling in an ice bath for 10 minutes. After cooling, 3.0 mL of butanol was added to each tube with stirring slowly under inversion and centrifuged at 4000 rpm for 5 minutes. The supernatant was used in the spectrophotometric reading at 535 nm (Shimadzu, UV-1800). The concentration of the thiobarbituric-malonaldehyde acid complex was calculated from the standard malonaldehyde (MDA) curve. Butanol was used as blank, BHT as positive control, and methanol as negative control.

### 2.6. Inhibitory Capacity on the Pancreatic Lipase Enzyme

The assay to determine the inhibitory capacity against pancreatic lipase was performed by spectrophotometric method with some modifications [29]. MC and MPMO were prepared at the concentration of 10 mg/mL in dimethyl sulfoxide (DMSO). From this solution, the assay was performed using a 10 g/L swine pancreatic lipase solution in 0.05 mol/L Tris-HCl buffer, pH 8.0, containing 0.010 mol/L calcium chloride and 0.025 mol/L sodium chloride. The substrate *p*-nitrophenol palmitate (8 mmol/L) was dissolved in 0.5% Triton X-100. In triplicate, 50 *μ*L of the sample solution, 100 *μ*L of the enzyme, and 50 *μ*L of the substrate were placed into microtubes and incubated in a 37°C water bath at the times of 10, 20, 30, and 40 minutes. After this period, the reaction was stopped with an ice bath and 1.0 mL of 0.05 mol/L Tris-HCl buffer. For each time, the controls were used without enzyme (substrate blank) and without substrate (enzyme blank). As a positive control, 1 mg/mL orlistat was used. The absorbances of lipase products (*p*-nitrophenol) were determined using a spectrophotometer (Shimadzu, UV-1800) at 410 nm. After the absorbances were obtained, linear regression analysis by the least-squares method was performed to acquire the straight equation and angular coefficients (slope of the line), and inhibition of pancreatic lipase was determined. The percent inhibition (% *I*) of pancreatic lipase was calculated according to the following equation:

(3) $$\begin{array}{c} \%I=100\times \frac{\left(A-a\right)–\left(B-b\right)}{\left(A-a\right)}, \end{array}$$

where *A* is the absorbance in the absence of the possible inhibitor, which corresponds to the control enzyme assay; *a* is the absorbance in the absence of the sample and enzyme (blank substrate); *B* is the absorbance in the presence of the possible inhibitor with the enzyme and substrate; and *b* is the absorbance in the absence of the enzyme.

### 2.7. Molecular Docking Study

The three-dimensional structure of the ligands was generated in the MarvinSketch 16.7.4 program [30]. Then, the geometry of ligands was refined by semiempirical calculations using Parametric Method 7 (PM7) [31] implemented in MOPAC2012 software using the *Octopus* workflow [32]. The crystallographic coordinates of the three-dimensional structure of the protein were obtained from the Protein Data Bank (PDB) under code 1LPA for pancreatic lipase [33]. The validation of the crystallographic ligands obtained from PDB was done by a redocking procedure that consisted of reproducing a crystallographic protein-binder complex with root-mean-square deviation (RMSD) of less than 2 Å.

The molecular docking was performed by AutoDock Vina 1.1.2 program [34]. In addition, a grid box was generated with dimensions of 30 × 30 × 30 Å for molecular targets, and the coordinates of grid box were centered on crystallographic ligand with *x* 6.309, *y* 27.567, and *z* 48.586 Å using MGLTools software [35]. The analyses of the molecular recognition interactions were performed through the Discovery Studio v. 4.5 2016 program [36, 37].

### 2.8. Statistical Analyses

The results were expressed as mean ± standard error mean (SEM). Analysis of variance (ANOVA) followed by Tukey's HSD (honest significant difference) test was applied to measure the degree of significance for *P* < 0.05. The GraphPad Prism® program was used in these analyses.

## 3. Results

### 3.1. Synthesis of 2-[(4-Methoxyphenyl)methyl]oxirane

2-[(4-Methoxyphenyl)methyl]oxirane (MPMO) was synthesized from methyl chavicol, which showed the physical appearance of a brown oily liquid of molecular formula C_10_H_12_O_2_ and molecular mass 164.204 g·mol^−1^. The yield of the reaction was 75% with purity of 99% when analyzed by gas chromatography (GC) (Supplementary 7).

The spectral data obtained were the following: ^1^H NMR, 500 Hz, (CDCl_3_): *δ* (ppm): 6.85 (d, 2H, *J* = 2.14 Hz); 6.74 (d, 2H, *J* = 2.14 Hz); 3.89 (s, 3H, *J* = 5.53, 5.53, 3.89, 2.75 Hz); 2.70 (m, 3H); 2.56 (dd, 1H, *J* = 5.04, 2.59 Hz); and 2.11 (m, 3H) (Supplementary 4). ^13^C NMR, 75 Hz, (CDCl_3_): *δ* (ppm): 171.395; 145.782; 145.608; 130.553; 120.612; 115.412; 110.935; 60.610; 56.193; and 52.774 (Supplementary 5). MS: *m*/*z* = 164 (M^+^); 121; 108; 91; 77; and 65 (Supplementary 6).

### 3.2. DPPH Radical Sequestration Method

**Figure 1:**
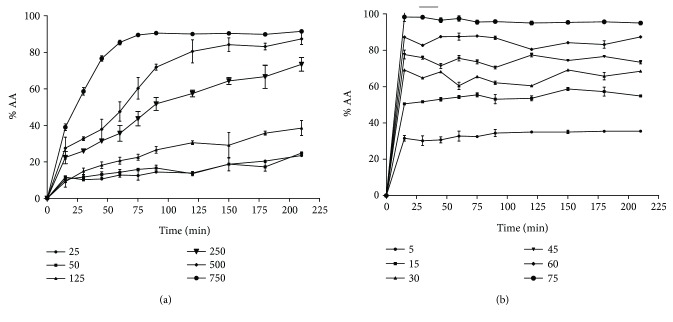
Kinetic profile of the methyl chavicol and 2-[(4-methoxyphenyl)methyl]oxirane at different concentrations (mg/mL) against DPPH. The values correspond to the mean ± SEM (*n* = 3). (a) Methyl chavicol (50 to 750 mg/mL); (b) 2-[(4-methoxyphenyl)methyl]oxirane (5 to 75 mg/mL).

The kinetic profile showed that MC has a slow antioxidant capacity with reaction time greater than one hour. The percentage of unreacted DPPH radicals with MC in relation to the time is shown in Figure 1. When the steady state was reached, about 90 minutes (methyl chavicol) and 15 minutes (2-[(4-methoxyphenyl)methyl]oxirane), the reaction between antioxidant and DPPH ceased. Thus, it was possible to calculate the real amount of DPPH radicals that was reduced by MC, avoiding the selection of an inadequate time interval in which the reaction still occurs.

The antioxidant potential of MC, MPMO, and BHT against DPPH is presented in Table 1. IC_50_ values of the samples ranged from 0.01 ± 0.01 to 312.50 ± 2.28 mg/mL and were significantly different from each other (*P* < 0.001). Considering the concentration of 50 mg/mL, MPMO was more effective than MC in inhibiting DPPH, since it produced an activity percentage (%) of approximately 80% of inhibition.

### 3.3. Cooxidation of the *β*-Carotene/Linoleic Acid Method

**Figure 2:**
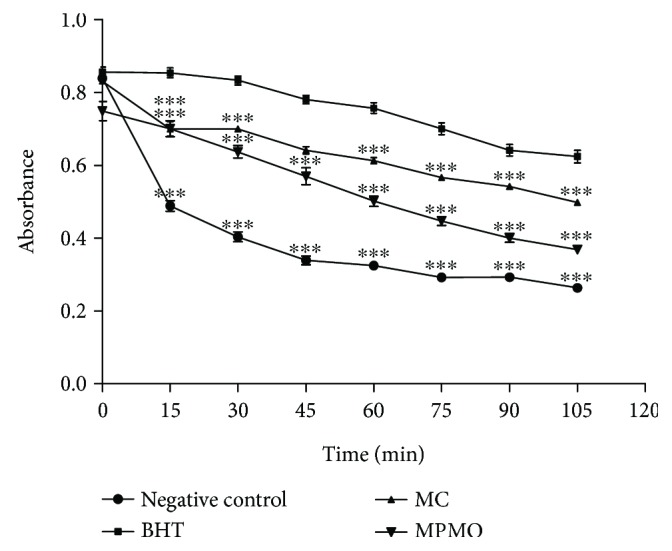
Decay of absorbance versus time by the cooxidation of the *β*-carotene/linoleic acid method. The values correspond to the mean ± SEM (*n* = 3). BHT: 3,5-di-*tert*-butyl-4-hydroxy toluene; MC: methyl chavicol; MPMO: 2-[(4-methoxyphenyl)methyl]oxirane. The means differed from those of the negative control after analysis of variance followed by Tukey's HSD (honest significant difference) test for ^∗∗∗^ *P* < 0.001.

Figure 2 shows the decay of the absorbances in relation to the time using the cooxidation of the *β*-carotene/linoleic acid method. MC was more effective in inhibiting lipid peroxidation, since this compound presented a lower decay when compared to MPMO. After 15 minutes, the absorbances of the compounds are different from those of the negative control (*P* < 0.001).

With the data in Table 2, one can observe that MC inhibited 73.08 ± 4.79% of the lipid peroxidation, while MPMO produced a reduction of 36.16 ± 4.11%. These data also show that MC was more effective than BHT (positive control) in the inhibition of lipid peroxidation.

### 3.4. Thiobarbituric Acid Method

The concentration of malonaldehyde (MDA) decreased in the homogenate treated with BHT, MC, and MPMO when compared to that of the negative control (Table 3). On the 5th day (day 4) of the experiment, MC presented an antioxidant activity similar to that of BHT in inhibiting the formation of MDA (*P* < 0.001).

### 3.5. Inhibitory Effect of the Methyl Chavicol and 2-[(4-Methoxyphenyl)methyl]oxirane on the Pancreatic Lipase

**Figure 3:**
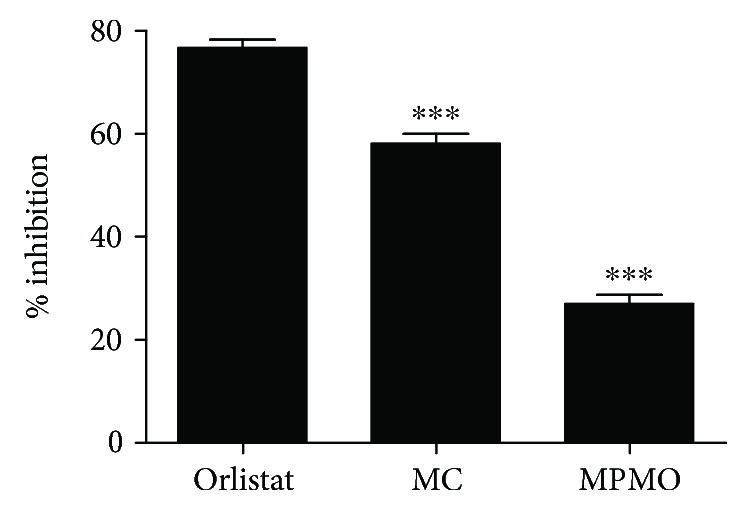
Inhibitory effect of the methyl chavicol and 2-[(4-methoxyphenyl)methyl]oxirane on the pancreatic lipase. The values correspond to the mean ± SEM (*n* = 3). MC: methyl chavicol (10 mg/mL); MPMO: 2-[(4-methoxyphenyl)methyl]oxirane (10 mg/mL). The means differed from those of the positive control (orlistat) after analysis of variance followed by Tukey's HSD (honest significant difference) test for ^∗∗∗^ *P* < 0.001.

The inhibitory activity of MC and MPMO on the pancreatic lipase was 58.12 and 26.93%, respectively. Orlistat, the positive control, was effective by 76.80% of inhibition (Figure 3).

### 3.6. Molecular Docking Study

In this investigation, the parameters were validated using the redocking method to reproduce a protein-ligand crystallographic complex with a root-mean-square deviation (RMSD) of less than 2 Å. The redocking of the crystallographic ligand, diundecylphosphatidylcholine (PLC) (1.3232 Å) and orlistat (1.84 Å) (Supplementary 8), at the pancreatic lipase binding site showed an expressive reconstruction of the crystallographic complexes, which was essential to conduct this study. From these data, the molecular docking on the pancreatic lipase (PDB 1LPA) (Supplementary 9) was performed to obtain the orientation of the ligands. The amino acid residues Ser153, Asp177, and His264, components of the catalytic triad, constituted the most important molecular interactions, and Ser153 was the main amino acid involved in the lipolysis. Van der Waals and hydrogen bond interactions are associated with Ser153 and His264 residues, respectively, possible targets of inhibition for the antilipase agents (Supplementary 9).

The molecular interactions between MC or MPMO and pancreatic lipase are of hydrogen bonding type that exhibited Ser153 and His264 residues as target amino acids (Supplementary 9). The molecular docking study also revealed that the effect of MC and MPMO on the lipase produced a binding affinity value equal to −6.1 kcal·mol^−1^, which was greater than that of orlistat (−6.5 kcal·mol^−1^) and lower when compared to that of PLC (−5.6 kcal·mol^−1^) (Table 4).

## 4. Discussion

**Scheme 2:**
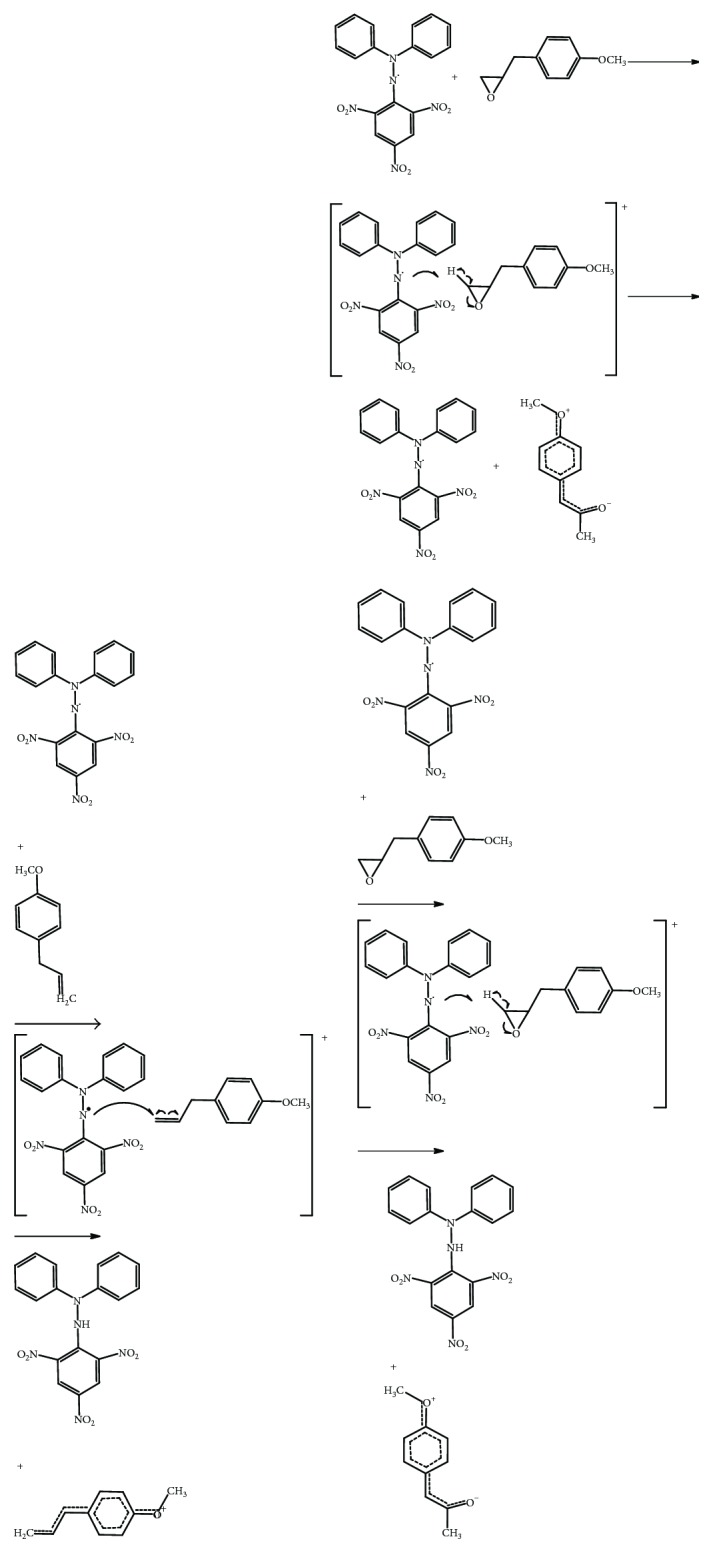
Proposal of the antioxidant mechanism of the methyl chavicol and 2-[(4-methoxyphenyl)methyl]oxirane based on the reaction with DPPH. (a) Stages of reactions between methyl chavicol and DPPH. (b) Stages of reactions between 2-[(4-methoxyphenyl)methyl]oxirane and DPPH.

MPMO synthesis consisted of an epoxidation in the olefinic group, since the epoxides are versatile and provide chirality to the molecules. Epoxides are also susceptible to reactions with a large number of nucleophiles, electrophiles, acids, and bases, with reducing agents and some oxidizing due to ring tension and polarity [38]. In this synthesis, the Prislaschajew reaction was carried out using the *meta*-chloroperbenzoic acid as an epoxidizing reagent. In addition, the structure of MC allows the functionalization of the olefin with peracids and occurs by a possible electrophilic biomolecular mechanism, where the peracid would be in a cyclic structure, stabilized by an intramolecular hydrogen bond to form a chelate (Scheme 2). The main evidence for this mechanism is the increase in the reaction rate due to the presence of electron-withdrawing substituents in the peracid. This fact increases the electrophilicity of O–O bond and/or the presence of electron donor groups that raise the nucleophilicity of C–C bond. The electrophilic mechanism is reinforced by the basicity of the solvent, breaking the intramolecular hydrogen bond, which decelerates the reaction. In this sense, the epoxidation of olefins with peracids is generally carried out in low polar aprotic solvents such as dichloromethane [38].

The results of the antioxidant activity showed that MC and MPMO presented an antioxidant potential in trials that differ in relation to the evaluated mechanism. In the DPPH free radical sequestration assay, the IC_50_ value of MC (312.50 ± 2.28 mg/mL) was about 38 times lower than that of MPMO (8.29 ± 0.80 mg/mL). The presence of the epoxide in MPMO can justify the higher antioxidant action when compared to MC, since this group increases its polarity and allows the electron donation to the DPPH radical. In addition, components of essential oils containing hydroxyl groups attached to the aromatic ring, unsaturations, and availability of electrons are associated with the antioxidant activity [39]. However, the essential oil of *Tagetes lucida*, which contains 95.7% of MC, produced IC_50_ of 37.9 *μ*g/mL [40], while sweet basil essential oil (17.06% of MC) showed IC_50_ of 1.092 ± 0.066 mg/mL [41]. Probably, this difference is related to compounds that may promote synergistic action among them [41, 42].

Lipid peroxidation by the cooxidation of *β*-carotene/linoleic acid system and thiobarbituric acid assays are *in vitro* tests that reproduce physiological situations of oxidative stress, which can lead to cell death in extreme cases [43, 44]. As observed in the decay of the absorbance plot as a function of time (Figure 2), MC and MPMO delayed and reduced lipoperoxidation and, consequently, the oxidation of *β*-carotene. Thus, these findings show that MC is a molecule with antioxidant potential against lipid peroxidation, since it inhibited 73.08% of the oxidative process, while BHT (positive control) presented 59.66%, and MPMO was able to inhibit 36.16%. In addition, the results of the thiobarbituric acid assay corroborate the data on cooxidation of *β*-carotene/linoleic acid system, since MC was more active in inhibiting the formation of malonaldehyde in the homogenate. Lipoperoxidation is a process that involves the initiation, propagation, and termination steps, and antioxidants can block the first step (initiation) by neutralizing reactive oxygen species and/or inhibiting the propagation by suppressing the peroxyl radicals [44, 45]. Probably, MC reduced the generation of lipoperoxides by decreasing the formation of chelate complexes with reduced metals. Because it is a compound with lower polarity, MC has higher affinity for lipid media than has MPMO. In addition, unlike the epoxide group in the MPMO structure, the presence of the olefin in MC reduces the polarity and allows a greater lipid peroxidation inhibition.

The evaluation of the antilipase activity of MC is based on the kinetics of the lipolytic hydrolysis reaction of the *p*-nitrophenol palmitate in palmitic acid and *p*-nitrophenol. The release of these substances results in a yellow-colored chromogen that is intensified at high pH (optimum pH equals 8) [46]. A surfactant, such as Triton X-100, increases lipophilicity and stabilizes the reaction medium as well as enhances the permeability. Salts such as NaCl and CaCl_2_ decrease the solvation layer of the enzyme, which implies a better dissolution, increases the ionic strength of the medium, and enables the formation of the ligand-protein complex [29]. Our results show that MC was more effective than MPMO, since MC was able to inhibit the *in vitro* pancreatic lipase by 58.12% (Figure 3), and these data were corroborated by the molecular docking study (Supplementary 9). Although Ser153 is the most important amino acid involved in the lipolysis, the pancreatic lipase inhibition occurs at the catalytic triad containing Ser153, Asp177, and His264 residues [47]. Both MC and MPMO interacted with Ser153 through a hydrogen bond, while the crystallographic binder (PLC) interacted with Ser153 by means of van der Waals force and His264 through a hydrogen bond. The formation of an electrostatic interaction between His264 and carbonyl oxygen differentiated the orlistat action of the other ligands at the catalytic site. It is important to note that a hydrogen bond interaction is typically more stable than a van der Waals force [48]. These findings also showed that MC and MPMO were less active than orlistat but were more active than PLC, since they produced a lower-affinity energy value (Table 4). Furthermore, it was verified that the oxygen of the epoxide found in MPMO was able to maintain a hydrogen bond at the Ser153 residue, but there was no interaction with the other residues responsible for its lipolytic action.

Natural compounds, such as alkaloids, carotenoids, glycosides, polyphenols, polysaccharides, saponins, and terpenoids, are described as pancreatic lipase inhibitors [48]. In particular, terpenes, such as carnosic acid, carnosol, roylenoic acid, 7-methoxyrosmanol, and oleanolic acid, were reported to inhibit pancreatic lipase [49, 50]. Considering these aspects, MC and its synthetic analogue MPMO may constitute a new class of antilipase agents belonging to the phenylpropanoid derivatives.

Although methyl chavicol is found in essential oils of medicinal plants widely used by the population [43], the carcinogenic and teratogenic effects should be considered in possible therapeutic applications. Estragole and its metabolite 1′-hydroxyestragole, for example, induced hepatic tumors in mice either after dietary chronic exposure or after intraperitoneal or subcutaneous injections [12–14]. In addition, the electrophilic epoxides of estragole and 1′-hydroxyestragole are directly mutagenic in *S. typhimurium*. Both estragole and its 1′-hydroxy metabolite produced unscheduled DNA synthesis in rat hepatocytes *in vitro* and estragole also *in vivo*. The formation of hepatic DNA adducts has also been demonstrated in mice. In this sense, the toxicity of methyl chavicol and its analogue with generation of toxic metabolites [12–15] may prevent their therapeutic use as antioxidant and antilipase agents.

## 5. Conclusion

MC and MPMO have an antioxidant activity and are capable of inhibiting the pancreatic lipase enzyme using the *in vitro* and in silico assays. The results suggest that these compounds may be promising for the development of new therapeutic options for the treatment of diseases associated with oxidative processes and metabolic alterations.

## Figures and Tables

**Table 1:** IC_50_ values of the methyl chavicol and 2-[(4-methoxyphenyl)methyl]oxirane by the DPPH method.

Compound	IC_50_ (mg/mL)
BHT	0.01 ± 0.01
MC	312.50 ± 2.28^∗∗∗^
MPMO	8.29 ± 0.80^∗∗∗^

The values correspond to the mean ± SEM (*n* = 3). BHT: 3,5-di-*tert*-butyl-4-hydroxy toluene; MC: methyl chavicol; MPMO: 2-[(4-methoxyphenyl)methyl]oxirane. The means differed from those of the positive control (BHT) after analysis of variance followed by Tukey's HSD (honest significant difference) test for ^∗∗∗^ *P* < 0.001.

**Table 2:** Inhibition of lipid peroxidation by the cooxidation of the *β*-carotene/linoleic acid method.

Compound	Inhibition of lipid peroxidation (%)
BHT	59.66 ± 0.52
MC	73.08 ± 4.79^∗∗∗^
MPMO	36.16 ± 4.11^∗∗∗^

The values correspond to the mean ± SEM (*n* = 3). BHT: 3,5-di-*tert*-butyl-4-hydroxy toluene; MC: methyl chavicol; MPMO: 2-[(4-methoxyphenyl)methyl]oxirane. The means differed from those of the positive control (BHT) after analysis of variance followed by Tukey's HSD (honest significant difference) test for ^∗∗∗^ *P* < 0.001.

**Table 3:** Concentration of malonaldehyde (MDA) obtained by the thiobarbituric acid method.

Sample	Concentration (mg)	MDA content (*μ*M)				
		Day 0	Day 1	Day 2	Day 3	Day 4
Control	Saline	0.42 ± 0.02	0.59 ± 0.04	0.67 ± 0.06	0.64 ± 0.03	0.91 ± 0.08

BHT	7.5	0.08 ± 0.02^∗∗∗^	0.16 ± 0.02^∗∗∗^	0.12 ± 0.03^∗∗∗^	0.16 ± 0.00^∗∗∗^	0.22 ± 0.05^∗∗∗^
	15	0.10 ± 0.02^∗∗∗^	0.11 ± 0.03^∗∗∗^	0.08 ± 0.01^∗∗∗^	0.17 ± 0.06^∗∗∗^	0.16 ± 0.01^∗∗∗^
	30	0.20 ± 0.01^∗∗∗^	0.20 ± 0.03^∗∗∗^	0.23 ± 0.06^∗∗∗^	0.12 ± 0.08^∗∗∗^	0.22 ± 0.08^∗∗∗^

MC	7.5	0.41 ± 0.01	0.18 ± 0.02^∗∗∗^	0.22 ± 0.06^∗∗∗^	0.22 ± 0.06^∗∗∗^	0.16 ± 0.02^∗∗∗^
	15	0.39 ± 0.06	0.29 ± 0.04^∗∗∗^	0.53 ± 0.06^∗∗∗^	0.32 ± 0.07^∗∗∗^	0.21 ± 0.08^∗∗∗^
	30	0.35 ± 0.02^∗∗∗^	0.20 ± 0.01^∗∗∗^	0.40 ± 0.06^∗∗∗^	0.38 ± 0.03^∗∗∗^	0.22 ± 0.02^∗∗∗^

MPMO	7.5	0.24 ± 0.02^∗∗∗^	0.39 ± 0.07^∗∗∗^	0.46 ± 0.01^∗∗∗^	0.35 ± 0.03^∗∗∗^	0.54 ± 0.04^∗∗∗^
	15	0.20 ± 0.01^∗∗∗^	0.25 ± 0.00^∗∗∗^	0.37 ± 0.05^∗∗∗^	0.39 ± 0.01^∗∗∗^	0.38 ± 0.01^∗∗∗^
	30	0.18 ± 0.06^∗∗∗^	0.26 ± 0.02^∗∗∗^	0.32 ± 0.00^∗∗∗^	0.37 ± 0.01^∗∗∗^	0.34 ± 0.05^∗∗∗^

The values correspond to the mean ± SEM (*n* = 3). BHT: 3,5-di-*tert*-butyl-4-hydroxy toluene; MC: methyl chavicol; MPMO: 2-[(4-methoxyphenyl)methyl]oxirane. The means differed from those of the negative control (saline) after analysis of variance followed by Tukey's HSD (honest significant difference) test for ^∗∗∗^ *P* < 0.001.

**Table 4:** Binding affinity between ligands and pancreatic lipase.

Compound	Binding affinity (kcal·mol^−1^)
PLC	−5.6
Orlistat	−6.5
MC	−6.1
MPMO	−6.1

PLC: diundecylphosphatidylcholine; MC: methyl chavicol; MPMO: 2-[(4-methoxyphenyl)methyl]oxirane.

## Supplementary Materials

Supplementary Materials Supplementary 1: ^1^HNMR spectrum of the methyl chavicol. Supplementary 2: ^13^CNMR spectrum of the methyl chavicol. Supplementary 3: mass spectrum of the methyl chavicol. Supplementary 4: ^1^HNMR spectrum of the 2-[(4-methoxyphenyl) methyl]oxirane. Supplementary 5: ^13^CNMR spectrum of the 2-[(4-methoxyphenyl)methyl]oxirane. Supplementary 6: mass spectrum of the 2-[(4-methoxyphenyl)methyl]oxirane. Supplementary 7: physicochemical properties and spectral data of the 2-[(4-methoxyphenyl)methyl]oxirane. Supplementary 8: redocking of the crystallographic ligands PLC and orlistat with RMSD 1.3232 Å and 1.84 Å, respectively.

## References

[1] Sosa V., Moliné T., Somoza R., Paciucci R., Kondoh H., LLeonart M. E. (2013). Oxidative stress and cancer: an overview. *Ageing Research Reviews*.

[2] Valko M., Leibfritz D., Moncol J., Cronin M. T. D., Mazur M., Telser J. (2007). Free radicals and antioxidants in normal physiological functions and human disease. *The International Journal of Biochemistry & Cell Biology*.

[3] Picon P. X., Zanatta C. M., Gerchman F., Zelmanovitz T., Gross J. L., Canani L. H. (2006). Análise dos critérios de definição da Sídrome Metabólica em Pacientes com Diabetes Metilo tipo 2. *Arquivos Brasileiros Endocrinologia & Metabologia*.

[4] Mattson M. P. (2008). Dietary factors, hormesis and health. *Ageing Research Reviews*.

[5] Li L., Ishdorj G., Gibson S. B. (2012). Reactive oxygen species regulation of autophagy in cancer: implications for cancer treatment. *Free Radical Biology & Medicine*.

[6] Magalhães L. M., Segundo M. A., Reis S., Lima J. L. F. C. (2008). Methodological aspects about *in vitro* evaluation of antioxidant properties. *Analytica Chimica Acta*.

[7] Paula J. P., Farago P. V., Ribas J. L. C. (2007). *In vivo* evaluation of the mutagenic potencial of estragole and eugenol chemotypes of *Ocimum selloi* Benth. essential oil. *Latin American Journal of Pharmacy*.

[8] de Paula J. P., Gomes-Carneiro M. R., Paumgartten F. J. R. (2003). Chemical composition, toxicity and mosquito repellency of *Ocimum selloi* oil. *Journal of Ethnopharmacology*.

[9] Silva-Alves K. S., Ferreira-da-Silva F. W., Peixoto-Neves D. (2013). Estragole blocks neuronal excitability by direct inhibition of Na^+^ channels. *Brazilian Journal of Medical and Biological Research*.

[10] de Souza Silva-Comar F. M., Wiirzler L. A. M., Silva-Filho S. E. (2014). Effect of estragole on leukocyte behavior and phagocytic activity of macrophages. *Evidence-based Complementary and Alternative Medicine*.

[11] Pattnaik S., Subramanyam V. R., Bapaji M., Kole C. R. (1997). Antibacterial and antifungal activity of aromatic constituents of essential oils. *Microbios*.

[12] Drinkwater N. R., Miller E. C., Miller J. A., Pitot H. C. (1976). Hepatocarcinogenicity of estragole (1-allyl-4-methoxybenzene) and 1′-hydroxyestragole in the mouse and mutagenicity of 1′-acetoxyestragole in bacteria. *Journal of the National Cancer Institute*.

[13] Miller E. C., Swanson A. B., Phillips D. H., Fletcher T. L., Liem A., Miller J. A. (1983). Structure-activity studies of the carcinogenicities in the mouse and rat of some naturally occurring and synthetic alkenylbenzene derivatives related to safrole and estragole. *Cancer Research*.

[14] Wiseman R. W., Fennell T. R., Miller J. A., Miller E. C. (1985). Further characterization of the DNA adducts formed by electrophilic esters of the hepatocarcinogens 1′-hydroxysafrole and 1′-hydroxyestragole in vitro and in mouse liver in vivo, including new adducts at C-8 and N-7 of guanine residues. *Cancer Research*.

[15] Jeurissen S. M. F., Punt A., Boersma M. G. (2007). Human cytochrome P450 enzyme specificity for the bioactivation of estragole and related alkenylbenzenes. *Chemical Research in Toxicology*.

[16] Anthony A., Caldwell J., Hutt A. J., Smith R. L. (1987). Metabolism of estragole in rat and mouse and influence of dose size on excretion of the proximate carcinogen 1′-hydroxyestragole. *Food and Chemical Toxicology*.

[17] Guenthner T. M., Luo G. (2001). Investigation of the role of the 2′,3′-epoxidation pathway in the bioactivation and genotoxicity of dietary allylbenzene analogs. *Toxicology*.

[18] Luo G., Qato M. K., Guenthner T. M. (1992). Hydrolysis of the 2′,3′-allylic epoxides of allylbenzene, estragole, eugenol, and safrole by both microsomal and cytosolic epoxide hydrolases. *Drug Metabolism and Disposition*.

[19] Phillips D. H., Miller J. A., Miller E. C., Adams B. (1981). Structures of the DNA adducts formed in mouse liver after administration of the proximate hepatocarcinogen 1′-hydroxyestragole. *Cancer Research*.

[20] Sangster S. A., Caldwell J., Hutt A. J., Anthony A., Smith R. L. (1987). The metabolic disposition of [methoxy-^14^C]-labeled *trans*-anethole, estragole and *p*-propylanisole in human volunteers. *Xenobiotica*.

[21] Solheim E., Scheline R. R. (1973). Metabolism of alkenebenzene derivatives in the rat I. *p*-Methoxyallylbenzene (estragole) and *p*-methoxypropenylbenzene (anethole). *Xenobiotica*.

[22] Phillips D. H., Reddy M. V., Randerath K. (1984). ^32^P-Post-labelling analysis of DNA adducts formed in the livers of animals treated with safrole, estragole and other naturally-occurring alkenylbenzenes. II. Newborn male B6C3F1 mice. *Carcinogenesis*.

[23] Randerath K., Haglund R. E., Phillips D. H., Reddy M. V. (1984). ^32^P-Post-labelling analysis of DNA adducts formed in the livers of animals treated with safrole, estragole and other naturally-occurring alkenylbenzenes. I. Adult female CD-1 mice. *Carcinogenesis*.

[24] Mensor L. L., Menezes F. S., Leitão G. G. (2001). Screening of Brazilian plant extracts for antioxidant activity by the use of DPPH free radical method. *Phytotherapy Research*.

[25] Mishra K., Ojha H., Chaudhury N. K. (2012). Estimation of antiradical properties of antioxidants using DPPH assay: a critical review and results. *Food Chemistry*.

[26] Koleva I. I., van Beek T. A., Linssen J. P. H., de Groot A., Evstatieva L. N. (2002). Screening of plant extracts for antioxidant activity: a comparative study on three testing methods. *Phytochemical Analysis*.

[27] Zeb A., Ullah F. (2016). A simple spectrophotometric method for the determination of thiobarbituric acid reactive substances in fried fast foods. *Journal of Analytical Methods in Chemistry*.

[28] Buege J. A., Aust S. D. (1978). [30] Microsomal lipid peroxidation. *Methods in Enzymology*.

[29] de Souza S. P., Pereira L. L. S., Souza A. A., dos Santos C. D. (2011). Inhibition of pancreatic lipase by extracts of *Baccharis trimera* (Less.) DC., Asteraceae: evaluation of antinutrients and effect on glycosidases. *Revista Brasileira de Farmacognosia*.

[30] Mayo S. L., Olafson B. D., Goddard W. A. (1990). DREIDING: a generic force field for molecular simulations. *The Journal of Physical Chemistry*.

[31] Stewart J. J. P. (2013). Optimization of parameters for semiempirical methods VI: more modifications to the NDDO approximations and re-optimization of parameters. *Journal of Molecular Modeling*.

[32] Van Tilbeurgh H., Egloff M. P., Martinez C., Rugani N., Verger R., Cambillau C. (1993). Interfacial activation of the lipase-procolipase complex by mixed micelles revealed by X-ray crystallography. *Nature*.

[33] Maia E. H. B., Campos V. A., dos Reis Santos B. (2017). Octopus: a platform for the virtual high-throughput screening of a pool of compounds against a set of molecular targets. *Journal of Molecular Modeling*.

[34] Trott O., Olson A. J. (2010). AutoDock Vina: improving the speed and accuracy of docking with a new scoring function, efficient optimization, and multithreading. *Journal of Computational Chemistry*.

[35] de Oliveira M. E., Cenzi G., Nunes R. R. (2013). Antimalarial activity of 4-metoxychalcones: docking studies as falcipain/plasmepsin inhibitors, ADMET and lipophilic efficiency analysis to identify a putative oral lead candidate. *Molecules*.

[36] Morris G. M., Huey R., Lindstrom W. (2009). AutoDock4 and AutoDockTools4: automated docking with selective receptor flexibility. *Journal of Computational Chemistry*.

[37] Santos Junior M. C., Assis S. A., Góes-Neto A. (2013). Structure-based drug design studies of UDP-*N*-acetylglucosamine pyrophosphosrylase, a key enzyme for the control of witches’ broom disease. *Chemistry Central Journal*.

[38] Von Holleben M. L. A., Schuch C. M. (1996). Ativação do peróxido de hidrogênio para a epoxidação de olefinas não funcionalizadas. *Química Nova*.

[39] Amic D., Davidovic-Amic D., Beslo D., Rastija V., Lucic B., Trinajstic N. (2007). SAR and QSAR of the antioxidant activity of flavonoids. *Current Medicinal Chemistry*.

[40] Olivero-Verbel J., González-Cervera T., Güette-Fernandez J., Jaramillo-Colorado B., Stashenko E. (2010). Chemical composition and antioxidant activity of essential oils isolated from Colombian plants. *Brazilian Journal of Pharmacognosy*.

[41] Li H., Ge Y., Luo Z. (2017). Evaluation of the chemical composition, antioxidant and anti-inflammatory activities of distillate and residue fractions of sweet basil essential oil. *Journal of Food Science and Technology*.

[42] Damien Dorman H. J., Deans S. G., Noble R. C., Surai P. (1995). Evaluation *in vitro* of plant essential oils as natural anti-oxidants. *Journal of Essential Oil Research*.

[43] Pisoschi A. M., Pop A. (2015). The role of antioxidants in the chemistry of oxidative stress: a review. *European Journal of Medicinal Chemistry*.

[44] Lima E. S., Abdalla D. S. P. (2010). Peroxidação lipídica: mecanismos e avaliação em amostras biológicas. *Revista Brasileira de Ciências Farmacêuticas*.

[45] Duarte-Almeida J. M., dos Santos R. J., Genovese M. I., Lajolo F. M. (2006). Avaliação da atividade antioxidante utilizando sistema *β*-caroteno/ácido linoleico e método de seqüestro de radicais DPPH. *Ciência e Tecnologia de Alimentos*.

[46] Teng Y., Xu Y. (2007). A modified *para*-nitrophenyl palmitate assay for lipase synthetic activity determination in organic solvent. *Analytical Biochemistry*.

[47] Lowe M. E. (1992). The catalytic site residues and interfacial binding of human pancreatic lipase. *The Journal of Biological Chemistry*.

[48] Veeramachaneni G. K., Raj K. K., Chalasani L. M., Annamraju S. K., Bondili J. S., Talluri V. R. (2015). Shape based virtual screening and molecular docking towards designing novel pancreatic lipase inhibitors. *Bioinformation*.

[49] Lunagariya N. A., Patel N. K., Jagtap S. C., Bhutani K. K. (2014). Inhibitors of pancreatic lipase: state of the art and clinical perspectives. *EXCLI Journal*.

[50] Ninomiya K., Matsuda H., Shimoda H. (2004). Carnosic acid, a new class of lipid absorption inhibitor from sage. *Bioorganic & Medicinal Chemistry Letters*.

