# Supplementary material for: Methyl Chavicol and Its Synthetic Analogue as Possible Antioxidant and Antilipase Agents Based on the In Vitro and In Silico Assays
Source: Oxid Med Cell Longev. 2018 Apr 11;2018:2189348. doi: 10.1155/2018/2189348 (PMC5925050; doi:10.1155/2018/2189348)
Supplement: Supplementary Materials — Supplementary 1: 1HNMR spectrum of the methyl chavicol. Supplementary 2: 13CNMR spectrum of the methyl chavicol. Supplementary 3: mass spectrum of the methyl chavicol. Supplementary 4: 1HNMR spectrum of the 2-[(4-methoxyphenyl) methyl]oxirane. Supplementary 5: 13CNMR spectrum of the 2-[(4-methoxyphenyl)methyl]oxirane. Supplementary 6: mass spectrum of the 2-[(4-methoxyphenyl)methyl]oxirane. Supplementary 7: physicochemical properties and spectral data of the 2-[(4-methoxyphenyl)methyl]oxirane. Supplementary 8: redocking of the crystallographic ligands PLC and orlistat with RMSD 1.3232 Å and 1.84 Å, respectively. [file 2189348.f1.pdf]

Supplementary 1 - $^{1}$HNMR spectrum of the methyl chavicol

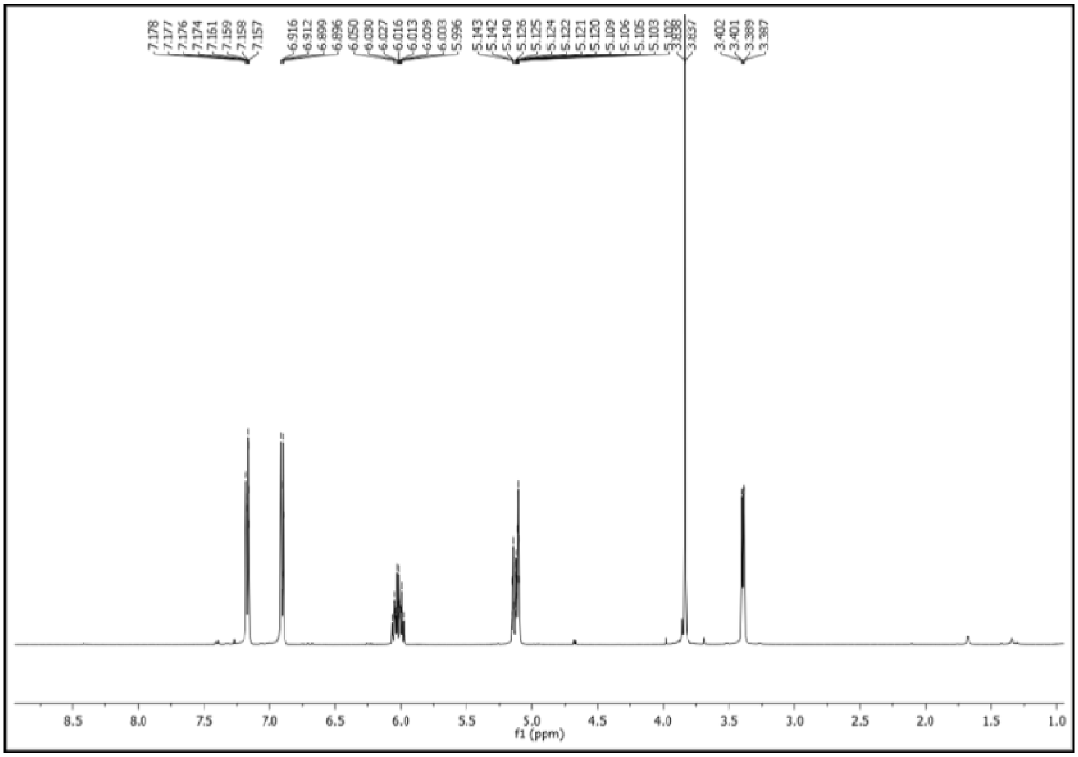

Supplementary 2 - $^{13}$CNMR spectrum of the methyl chavicol

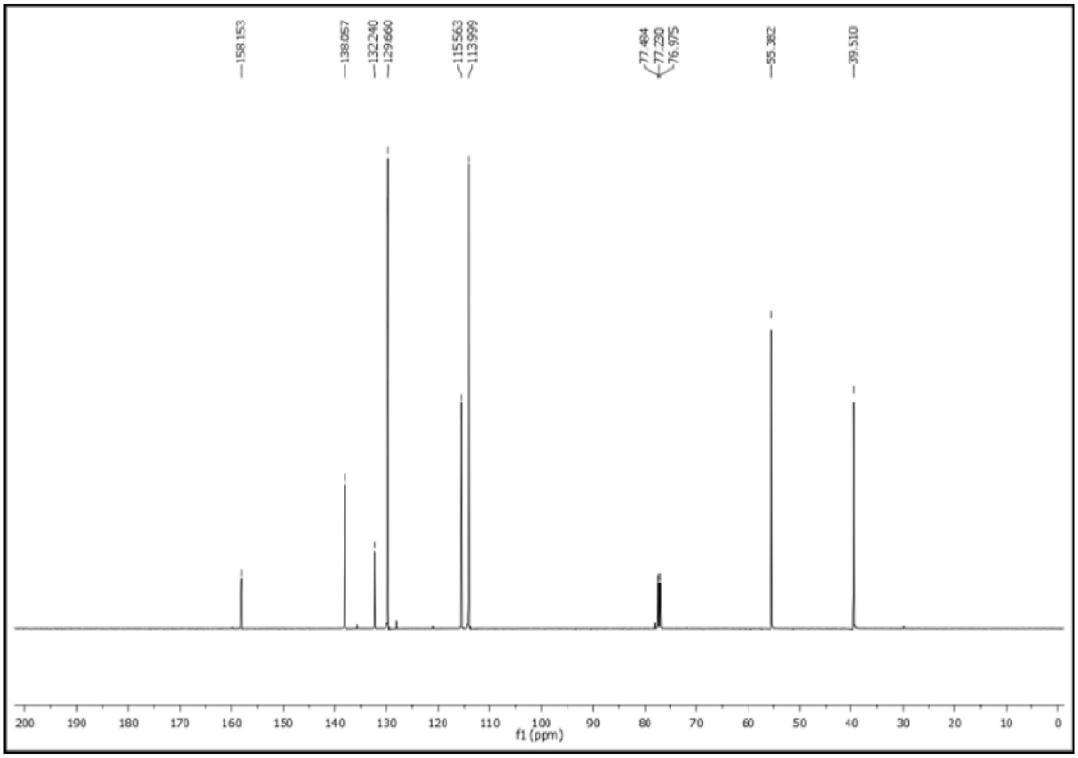

Supplementary 3 - Mass Spectrum of the methyl chavicol

<< Target >>
Line#:1 R.Time:9.355(Scan#:872) MassPeaks:166
RawMode:Averaged 9.350-9.360(871-873) BasePeak:148.15(7330228)
BG Mode:Calc. from Peak Group 1 - Event 1

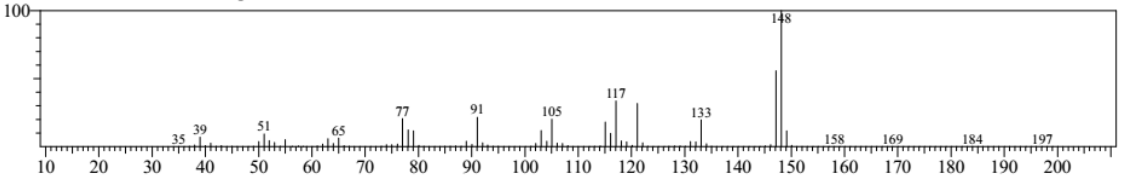

Supplementary 4 - $^{1}$HNMR spectrum of the 2-[(4-methoxyphenyl) methyl]oxirane

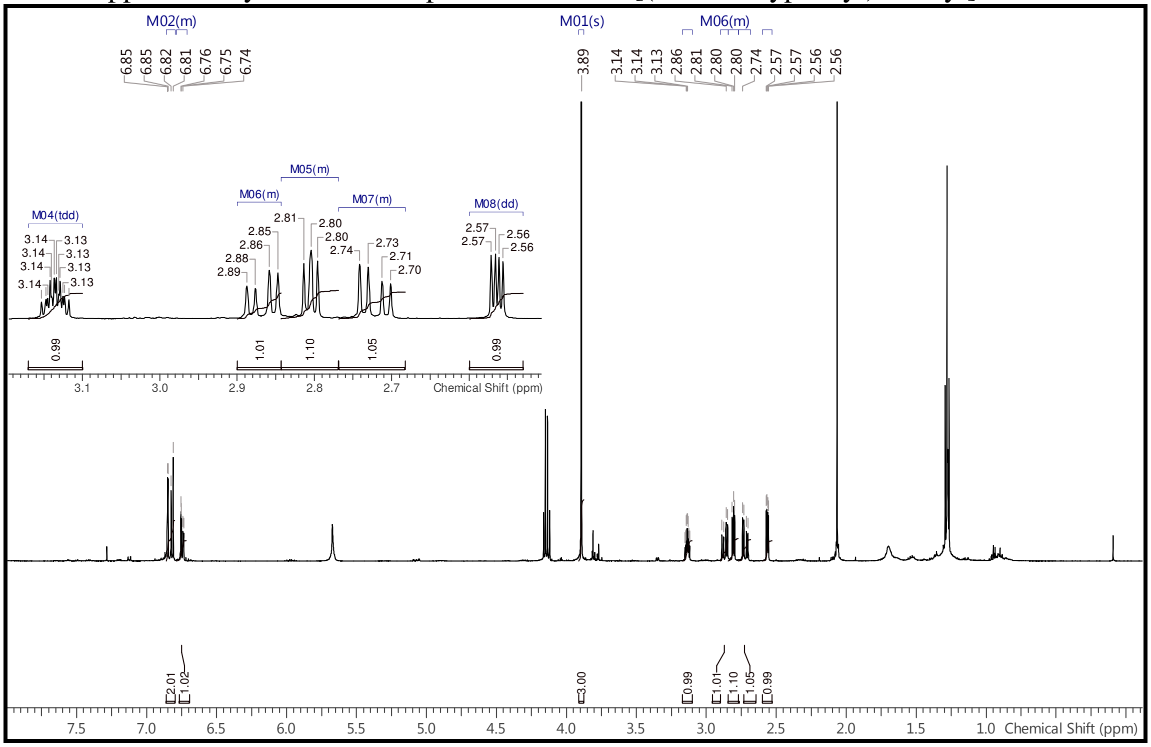

Supplementary 5 - $^{13}$CNMR spectrum of the 2-[(4-methoxyphenyl)methyl]oxirane

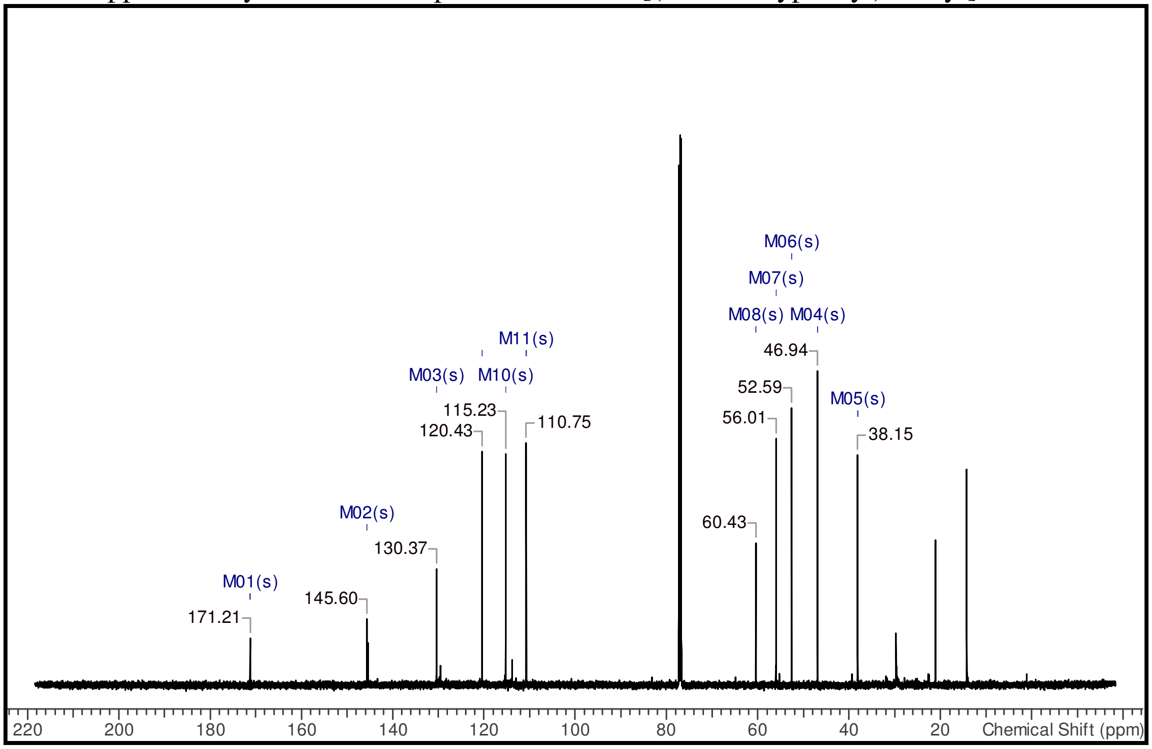

Supplementary 6 - Mass spectrum of the 2-[(4-methoxyphenyl)methyl]oxirane

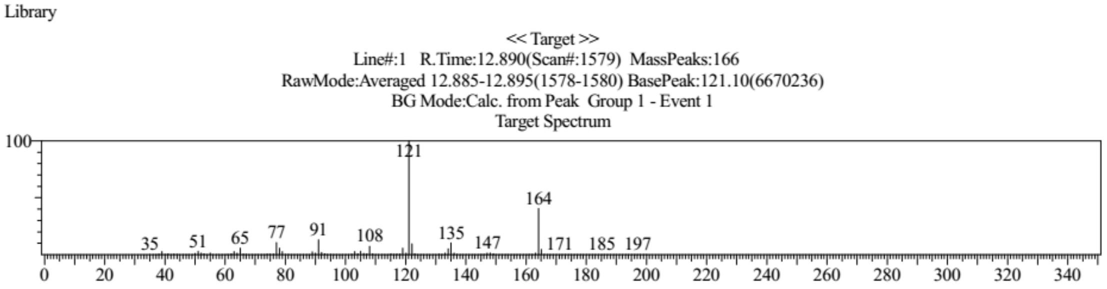

Supplementary 7 - Physico-chemical properties and spectral data of the 2-[(4-methoxyphenyl)methyl]oxirane

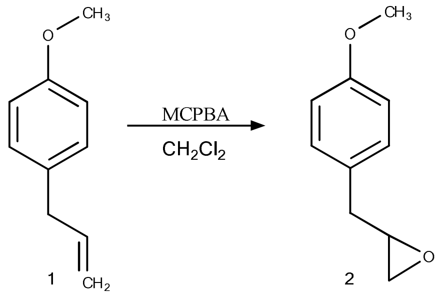


**Physical Appearance**: brown oily liquid

**M.F.:** $C_{10}H_{12}O_2$

**M.M.:** 164.204 $g.mol^{-1}$

**Yield**: 75%

**Purity (GC):** 99%

**$^1$HRMN, 500Hz, ($CDCl_3$): δ (ppm):** 6.85 (d, 2H, J = 2.14 Hz); 6.74 (d, 2H, J = 2.14 Hz); 3.89 (s, 3H); 3.14 (tdd, 1H, J = 5.53, 5.53, 3.89, 2.75 Hz); 2.70 (m, 3H); 2.56 (dd, 1H, J = 5.04, 2,59 Hz) and 2.11 (m, 3H).

**$^{13}$CRMN, 75Hz, ($CDCl_3$): δ (ppm):** 171.395; 145.782; 145.608; 130.553; 120.612; 115.412; 110.935; 60.610; 56.193 and 52.774.

**MS: m/z =** 164 (M+);147; 135; 121; 108; 91; 77 and 65.

Supplementary 8 - Redocking of the crystallographic ligands PLC and orlistat with RMSD 1.3232 Å and 1.84 Å, respectively.

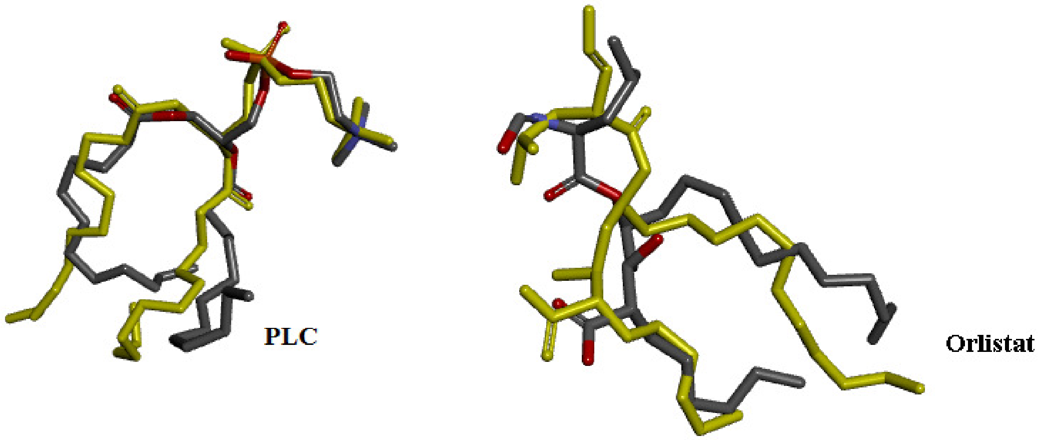


PLC: diundecylphosphatidylcholine

Supplementary 9 - Molecular orientations of crystallographic ligands at the pancreatic lipase binding site.

| Ligands | Molecular interactions between ligands and pancreatic lipase | |
|---|---|---|
| | Three-dimensional structure | Aminoacids residues that formed binding site and main interactions |
| PLC | 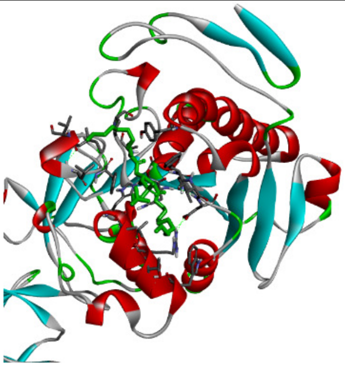 | 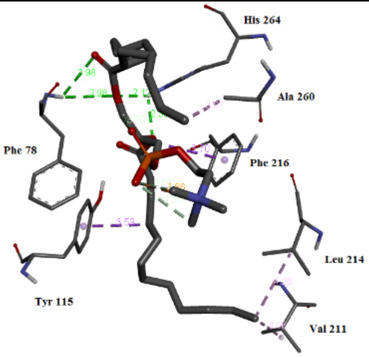  |
| Orlistat | 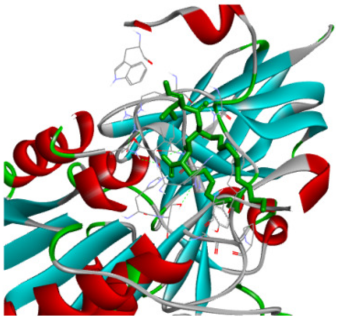 | 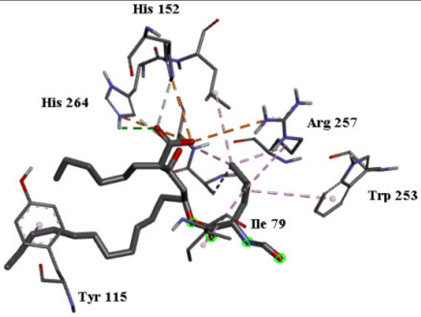  |
| MC | 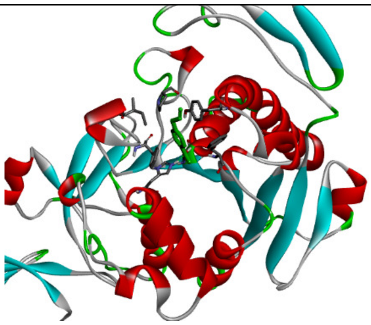 | 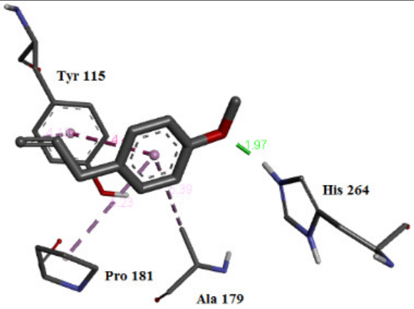  |
| MPMO | 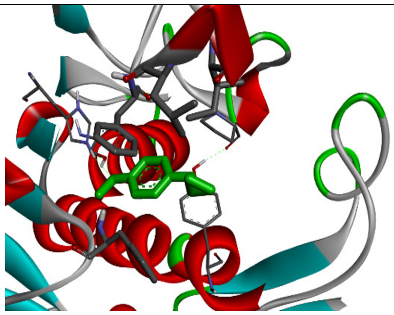 | 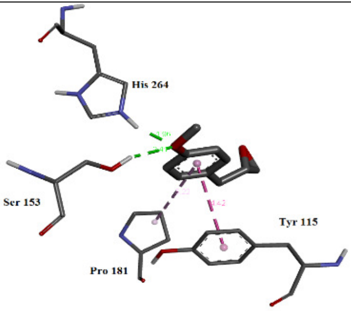  |

PLC: diundecylphosphatidylcholine; MC: methyl chavicol; MPMO: 2-[(4-methoxyphenyl)methyl]oxirane.
